# Working with clients engaging in recurrent suicidal behavior: impacts on community mental health practitioners

**DOI:** 10.3389/fpubh.2025.1537595

**Published:** 2025-03-13

**Authors:** Megan Collisson, Jacinta Hawgood

**Affiliations:** Australian Institute for Suicide Research and Prevention, World Health Organization Collaborating Centre for Research and Training in Suicide Prevention, School of Applied Psychology, Griffith University, Brisbane, QLD, Australia

**Keywords:** recurrent suicidal behavior, impacts of suicide, suicide prevention workforce, mental health workforce, systemic workplace issues

## Abstract

**Background:**

Practitioners have reported negative emotional impacts following client suicide. However, there is a paucity of literature exploring the impacts of working with clients engaging in suicidal behavior and on the impacts of repeat exposure to these events from the perspectives of practitioners.

**Aim:**

This qualitative study aimed to investigate the perceived impacts of working with clients engaging in recurrent suicidal behavior on Australian community mental health practitioners.

**Method:**

A phenomenological approach was utilized to explore the experiences of practitioners working with this client cohort; the impacts on them, the supports and resources used to sustain them and those perceived as necessary to increase the future sustainability of this important workforce. Eight practitioners were recruited via criterion snowball sampling to participate in semi-structured interviews up to a maximum of 45 min duration.

**Results:**

Participants were comprised of mental health clinicians (*N* = 6) and psychosocial support workers (*N* = 2) in community mental health settings. Thematic analysis revealed six major themes and several sub-themes. Practitioners experienced diversity of challenges and a diversity of emotional and psychological responses, along with impacts on their personal and professional lives. In response to these impacts, they reported positive experiences with individual supervision, debriefing and secondary consultation from specialist services, although access to these supports varied. Further, they highlighted the impact of systemic issues on their work with these clients including workforce shortages, staff turnover and client lack of access to needed supports (such as psychologists, psychosocial support workers and specialist services).

**Conclusion:**

While workplaces have a role to play in providing supports to reduce impacts on workers, further systemic work is required to increase sustainability of this workforce.

## 1 Introduction

Globally, more than 700,000 lives are lost to suicide each year, and the rate of suicide attempts is estimated to be around 20 times higher (1). In Australia in 2023 there were 3,214 lives were lost to suicide (2) and between 2021–2022, there were almost 24,800 hospitalizations for intentional self-harm (3). The impacts of both suicide and intentional self-harm are far-reaching, particularly in terms of emotional impacts on loved ones or those close to the person who took their life or experiencing suicidality (4, 5).

An often-forgotten population impacted by suicide and suicidal behavior is the clinical and non-clinical workforce who provide support, care and compassion to those experiencing suicidal distress. Despite studies over several decades highlighting the extent of emotional and psychological impacts on the mental health and suicide prevention workforce, very little has been done to respond to their mental health and well-being needs. Only recently in Australia, have several jurisdictions adopted a psychosocial code of practice which mandates workplaces to identify and reduce psychosocial hazards as far as is reasonably practical (6). Nevertheless, there have been no specific investigations exploring psychosocial hazards of the mental health workforce with respect to workers exposed to repeated client suicidal behavior and there has been no exploration of what supports might be needed from practitioners who work with these clients.

The suicide prevention and mental health workforce are frequently exposed to presentations of those experiencing suicidal distress and suicide. Studies have revealed that around 51% of health care professionals have experienced client suicide (7), while 76% of psychologists have experienced a client attempt suicide and 90.6% have worked with those experiencing suicidal distress (8). There remains a dearth of prevalence studies however providing more robust measurement of impacts of client suicide, suicidal behavior and especially recurrent suicidal behavior on the workforce.

Two recent systemic reviews explored the emotional impacts of client suicide on the worker (7, 9). These studies found that the most common reactions included sadness, guilt, shock and anger (7, 9). Practitioners also experienced helplessness, nightmares, sleep disruption, grief, distress and existential angst (7). Furthermore, professional impacts of client suicide on health care professionals included avoidance of suicidal clients, increasing the rate of hospitalization, more cautious practice, increased self-doubt and increased attention to legal aspects of practice (7). Health care professionals considered early retirement, with one health care practitioner retiring and some health care practitioners taking time off following the suicide (7). Health care professionals found that supervision, team meetings, counseling, case reviews, medical defense organizations and their own medical professionals were helpful (7). The experiences of support following client suicide varied significantly with between 11–87% of health care professionals across studies perceiving they received adequate support (7). This is an important finding, particularly considering that organizational and cultural factors along with availability of supports appeared to moderate the negative impacts of client suicide on workers (9).

However, there is limited literature on the experiences, emotional and psychological impacts on practitioners who work with clients engaging in suicidal behavior, with only five studies identified in this area (8, 10–13). No single study could be identified on the impacts of working with *recurrent* suicidal behavior, despite findings from a systemic review and meta-analysis that 16.3% of clients have subsequent admissions to the emergency department within 12 months of their index admission (14). With regards to specific diagnosis, a study completed in the outer eastern suburbs of Melbourne, Australia between 2015–2016 found that those with a diagnosis of borderline personality disorder were likely to represent to the emergency department with 73% representing in the audit year (15). The lack of research exploring the impacts of repeat exposure to these events prevents services and systems from adequately supporting practitioners.

All five studies exploring impacts of working with clients engaging in suicidal behavior (more generally) found negative emotional impacts of this work on practitioners (8, 10–13). Feelings of hopelessness and helplessness, anxiety, sadness, distress, anger and a sense of failure were revealed in a study comprised of surveys and follow-up interviews (12). However, there was no differentiation confirming whether these impacts occurred solely from client suicidal behavior, or whether they occurred after client suicide. A quantitative study utilizing the Impact of Event Scale, found increased levels of stress among master’s level (or higher) qualified social workers for working with suicidal behavior and from client suicide (10). Although, the study noted measures of avoidance and intrusion were significantly higher for those most impacted by client suicide (10). In a quantitative study comprised of multidisciplinary professionals including psychiatrists, psychologists and general practitioners, Rothes et al. (13) reported intrusion symptoms, distress, burnout and worker fear that the client would die by suicide. In another quantitative study, the most common reactions reported by psychologists working with clients engaging in suicidal behavior were anger, concern with death issues, guilt, sleep problems and emotional numbness (8). Finally, Kleespies et al. (11) found feelings of shock, fear, anger, sadness, sorrow for the client, discouragement, relief and a loss of self-confidence in pre-doctoral clinical psychology interns concerning their clients’ suicidal behavior. Interestingly, using the Impact of Event Scale, these authors also found no significant differences in stress levels of practitioners who had experienced a client suicide versus those who had experienced a client suicide attempt (11).

However, there are several limitations of these five-mentioned studies conducted to date. Noteworthy, Richards’ (12) study did not fully explore the impacts of working with suicidal behavior, rather focused primarily on transference and countertransference experiences of participants. While Kleespies et al. (11) explored some emotional impacts on practitioners using an initial telephone survey they did not undertake a more comprehensive qualitative investigation enabling rich and meaningful exploration of these experiences by interview as they did for pre-doctoral clinical psychology interns that had a client die by suicide. Furthermore, three studies (8, 10, 13) utilized quantitative methodology which did not allow for a full exploration of the subjective impacts on practitioners or exploration of their lived experience and the meaning attached to these experiences. The most recent of the five studies is now a decade old (13), with the earliest study now being over three decades old (11), which does not allow for meaningful understanding of practitioner experiences within contemporary suicide prevention and mental health workforces. Importantly, there was no comparison with practitioners who work with clients who have multiple suicidal presentations and or self-harming behaviors. As a result, the impacts of working with recurrent suicidal behavior within the suicide prevention and mental health workforce remain unknown.

Given the negative impacts of client suicide and suicidal behavior on mental health professionals outlined here, it could reasonably be assumed that workers exposed to recurrent or repeated client suicidal behavior, are at even higher risk of these negative impacts. Anecdotal reports from practitioners in Victoria, Australia, indicate that practitioners experience exhaustion and burnout from an under-resourced and crisis-driven mental health sector (16). More recently, over 200 senior psychiatrists in New South Wales, Australia have handed in their resignation citing safety concerns due to staffing shortages (17). These concerns have been rising as a result of staff turnover, crisis driven services and an under-funded community mental health sector (18). Despite these ongoing systemic issues and the known negative impacts of working with clients engaging in suicidal behavior, there has been little attention directed at the impacts on practitioners who work with repeat presentations of suicidal behavior. It is essential that these impacts are understood within the context of the necessity for broader reforms across the sector in order to provide support to practitioners and reduce staff turnover.

The primary aim of this study is to explore the impacts of working with clients engaging in recurrent suicidal behavior on the practitioner to enhance understanding of practitioner lived experience in this domain. A secondary aim is to explore supports and resources considered valuable or helpful for support and sustainability of practitioners in the mental health and suicide prevention workforce. To achieve these aims we posed the following questions:

- What are the impacts on practitioners working with clients engaging in recurrent suicidal behavior?
- What resources and/or supports are currently being accessed by practitioners working with clients engaging in recurrent suicidal behavior?
- How can practitioners working with clients engaging in recurrent suicidal behavior be supported in the workplace?

## 2 Methods

This study used a qualitative approach, using phenomenology to gain a deep and rich insight into the experiences of mental health practitioners in Australia. The study focused on exploring the lived experiences of practitioners working with clients engaging in recurrent suicidal behavior to understand the impacts on them, along with the supports they felt were necessary for the sustainability of their role in the suicide prevention and mental health workforce.

### 2.1 Participants, procedure, and design

The study adhered to Consolidated Criteria for Reporting Qualitative Research (COREQ) (19) (see Supplementary material 1 for COREQ checklist). Ethical approval was gained from the Griffith University Human Research Ethics Committee (GU Ref No: 2023/412). With the support of the Australian Institute for Suicide Research and Prevention (AISRAP), criterion snowball sampling was used to recruit participants via dissemination of advertisements on multiple social media platforms between June and July 2023. Eight potential participants responded to these online advertisements. Those who responded to advertisements were provided with participant information sheets which outlined the nature of the study, along with information about support services which could be contacted should the study result in any distress. After being provided with this information, participants were asked if they would like to proceed with the study. All eight respondents proceeded and none of these respondents withdrew at any time throughout the study. Participants were community mental health practitioners in Australia who had worked with a client who has engaged in suicidal behavior at least twice in a twelve-month period while under their care. Community mental health practitioners could involve a range of different healthcare professionals such as clinicians (occupational therapists, social workers, psychiatric nurses or psychologists), peer workers, psychosocial support workers and psychiatrists. These professionals work with clients outside of hospitals and mental health rehabilitation centers to provide longer-term support to clients who are in the community. Participants were entered into a prize draw to win one of three $50 gift cards for their participation and runners up were provided with a $25 gift card. Eight participants were interviewed for the study.

### 2.2 Data collection

Semi-structured interviews took place in July 2023 via Microsoft Teams at a mutually convenient time and lasted for a duration of 15–45 min. The interview began by obtaining verbal informed consent from practitioners. It then proceeded to open ended questions in line with the aims of the study – commencing with broader questions (e.g., to meet the primary aim: “What emotional, physical and/or psychological impacts (if any) have you noticed in this work?” and the secondary aim: “What supports (if any) do you access to sustain you in this work?”) and followed up with probing questions throughout the duration of the interview. No questions asked for personal information about the clients these practitioners worked with.

The individual interviews were conducted by lead author (M.C) via Microsoft Teams. Interviews were recorded and transcribed using Microsoft Teams software, and M.C went through each recording and transcript to ensure accuracy of verbatim quotes. Participants were able to choose their own alias at the time of the interview. The researcher collected demographic data at the beginning of the interview after obtaining verbal consent including age, gender, pronouns, highest level of education, length of time as a mental health practitioner, type of role employed as (e.g., clinician, peer, psychosocial support worker) and workplace setting (e.g., long-term community team, brief intervention).

### 2.3 Data analysis

An inductive approach was taken to analyze the data. Transcripts were initially reviewed by two coders (M.C, J.H) who made initial notes on transcripts. Data was then transferred to a Microsoft Excel spreadsheet where both coders noted initial reflections on each line of data separately in dedicated columns of the Microsoft Excel spreadsheet. These reflections were then reviewed by both coders together via multiple virtual meetings where themes were negotiated between the two researchers. The primary researcher (M.C) kept a reflective journal throughout the process.

### 2.4 Rigor

To increase reliability two coders were used in the coding process. The second author (J.H) initially coded half the interview data (transcripts 2, 4, 6 and 8). The lead author (M.C) coded all the data. After initially reviewing the data separately, the researchers came together via multiple virtual interviews to review each other’s initial reflections together. Through a process of negotiation all lines of data were reassessed together until consensus was reached on final themes/subthemes. A thematic map which outlined the themes and subsequent sub-themes was developed after the finalization of themes/subthemes and verbatim quotes are utilized in the findings to substantiate these themes/sub-themes. Both major and minor themes are presented in the results section. Interviews continued until data saturation appeared to be met at eight interviews. This became apparent through reflexive journaling and ongoing discussions between M.C and J.H.

The lead researcher (M.C) was a Master of Suicidology student at the time of the study who completed this study as part of her dissertation. At the time of the study, she was a practicing social worker and mental health clinician in public mental health in Victoria, Australia. Some participants were known to M.C through similar workforce networks. All those who responded to the advertisement were provided with a participant information statement that outlined the aims and purpose of the study before their interviews. Those who knew M.C from workforce networks were aware she was a social worker and mental health clinician in public mental health. The second researcher (J.H) is a clinical psychologist with over 25 years of experience in suicide-focused research and clinical practice. J.H has completed numerous qualitative studies related to suicidology over this time and was the dissertation supervisor of the lead author.

## 3 Results

### 3.1 Demographics of participants

The participants in this study were all women, aged between 29–45 years old. Two were psychosocial support workers, and six were clinicians. Five participants had post-graduate qualifications, one had an undergraduate degree, one had a diploma, and one had a certificate IV. Four practitioners had between 2–4 years of experience, two had between 5–9 years of experience, and two had more than 10 years of experience. Two participants worked in brief intervention, two worked in long-term community teams and two worked a mixture of both brief and long-term intervention.

### 3.2 Themes

**Figure 1:**
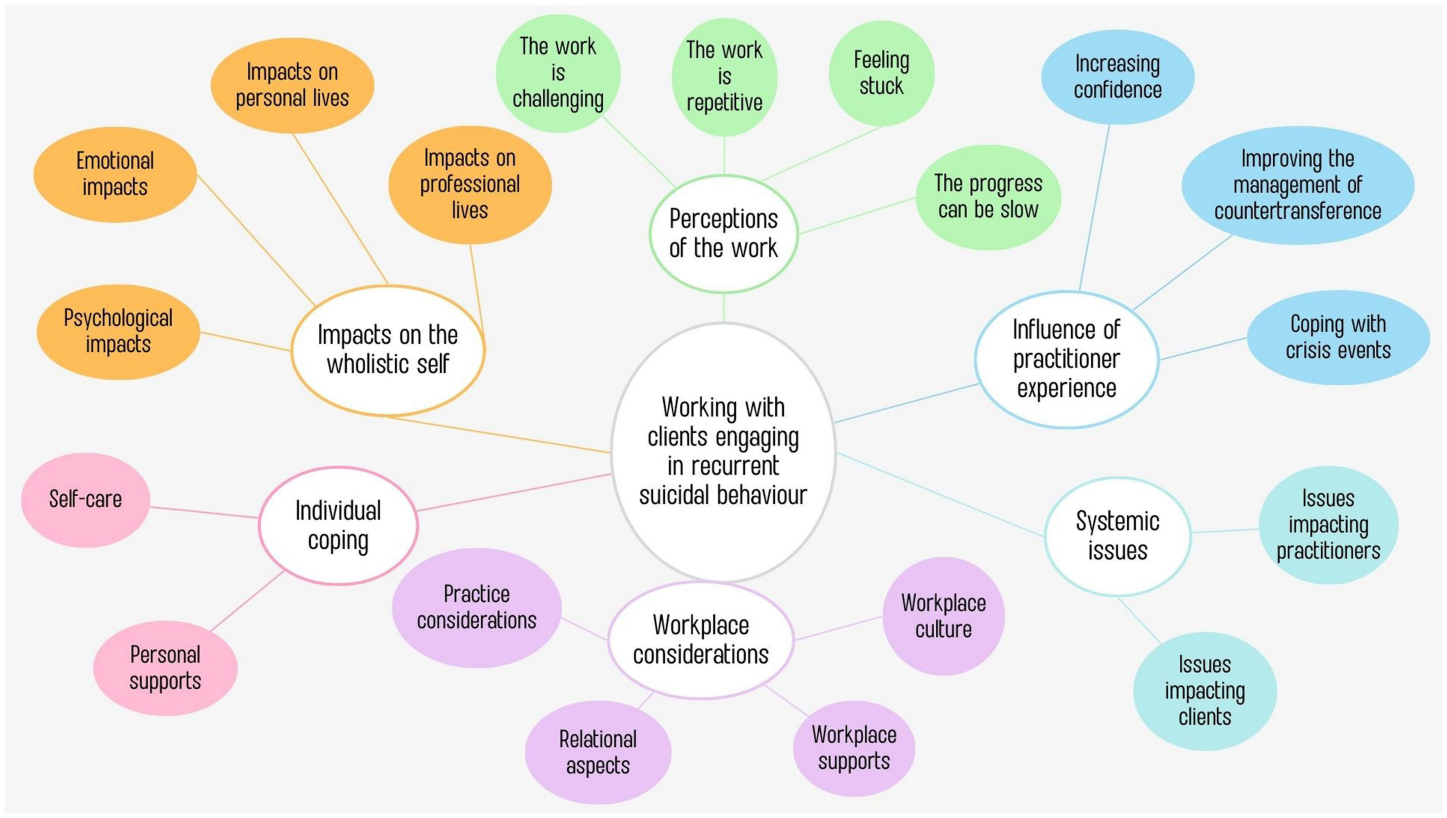
Thematic map of themes and subthemes.

As displayed in Figure 1, thematic analysis uncovered six major themes including: (1) impacts on the wholistic self; (2) perceptions of the work; (3) influence of practitioner experience; (4) individual coping; (5) workplace considerations; (6) systemic issues. Each theme also had several subthemes, also displayed in the thematic map (see Figure 1) and substantiated by verbatim quotes from participants which are conveyed in more detail subsequently.

### 3.3 Impacts on the wholistic self

The first theme focused on practitioners and the various impacts on their psychological, emotional wellbeing along with their personal and professional lives.

#### 3.3.1 Psychological impacts

Practitioners described feeling drained of energy, exhaustion and fatigue. This was captured by Sally who explains, “you feel fatigued, like that feeling of being drained of your energy and mentally full like you cannot bring on anymore.” Furthermore, practitioners reported hopelessness about the work and broader systems. Cynical feelings were described by Abby who expressed, “I’ve become more cynical about the mental health system and the world in general.” Kellie had dreams about the work, but it was not clear whether they were negative. Practitioners expressed feeling ‘on edge’ and activated while trying to prevent suicidal behavior as Kellie explained, “I think you are always prepared for something to happen… you are always in that activated state of mind.”

Mary described the impact on her confidence in her decision making, “you know, you are internally questioning yourself: am I good at this job? Am I doing the right things? Did I make the right decision about that [client]?.” Others, like Frankie, questioned their life decisions, “it gets to a point where it feels like: what am I doing with my life? Why am I doing this? What is the point?”

Taylor explained that she has not had any significant psychological impacts from this work, ‘I would not really say that I’ve had major psychological impact from it.” Furthermore, noticing the impacts on colleagues was noted by several practitioners as Molly articulates:

*I think it’s very good to consider and be mindful for us of the way that working with this kind of cohort can impact us emotionally, because I think that the focus as mental health clinicians that we have is on the emotional state and thoughts and feelings of our clients and we whilst in that mode, position ourselves as being objective observers, which obviously [we are] not objective or just observing we are participating, and I think that it’s much easier to see in my colleagues because I’m not them and I’m not in their brains at that point.*

#### 3.3.2 Emotional impacts

Anxiety and stress were noted as common impacts, however, Molly noted these were sometimes context-dependent with certain things influencing this such as the level of perceived risk:

*Depending on the context, it can be quite stressful, or it can be very day-to-day standard depending on how well the pattern of behaviour is known to me and the rest of the team and the level of risk, like the level of the lethality say of the means that they choose.*

Practitioners like Mary described the worry that recurrent suicidal behavior may become lethal, “the worry it may become fatal and then how do you navigate that to ensure it does not reach breaking point.” Frustration, irritability and anger were expressed, however, this appeared to be more broadly directed at other practitioners and the system rather than at individual clients. Several practitioners explained that their own lived experience can surface: including their own histories of suicidality and their past personal trauma. Dee notes:

*I guess there’s my own past traumas that can be exacerbated sometimes. Things you think that you have dealt with in your life and sometimes you are dealing with situations that are quite similar, so that can be a bit triggering.*

Multiple practitioners described the rewarding nature of the work as Molly explains:

*When you see improvements, that can be very, very rewarding because you have got that tension that you have held at the start and then if you can find ways to support those people to improve their lives and not be in that position anymore, that can be really nice.*

#### 3.3.3 Impacts on personal lives

Practitioners explained that this work could sometimes carry over into their personal lives in various ways. For example, one practitioner described carrying over ‘tension’ from the work, while others spoke about the impact on their personal relationships by having less capacity to deal with issues related to this work in their personal lives (e.g., with loved ones having a difficult time). Molly explained that she has become less reactive to friends in crisis noting, “I tend to be a lot more blasé about friends struggling because I’ve got this much more significant context in terms of my work setting.”

Some practitioners expressed the ability to switch off outside of work. However, other practitioners described taking the work home with them and having trouble motivating themselves to see friends and family. Dee noted that she withdraws entirely, “I become withdrawn in myself just so I’m able to mentally process what has happened that day or that week.” Similarly, Sally explained using rest as recovery with realizations that her work life-balance was impacted:

*You do spend a lot of your energy in the daytime, managing and working closely with these people so I do find that parts of your life do get disrupted, without you realising until much later.*

#### 3.3.4 Impact on professional lives

Practitioners felt their professional lives were impacted by this work. These impacts included being frustrated with work and at less experienced practitioners who question their decision making. Abby expressed anger toward other practitioners who do not hold the same complexity on their caseloads, “I tend to shut down and I cannot even talk to my colleagues because I’m so angry [at colleagues for not sharing the load].”

Several practitioners discussed changes to their work practices like keeping clients at a distance and seeking reassurance from others about their work. Furthermore, they expressed that perhaps they did not take others who were not engaging in recurrent suicidal behaviors as seriously as those who were. Abby explained that she will sometimes avoid working with clients engaging in recurrent suicidal behavior, “I would actually try to avoid working with people with that nonfatal suicidal behaviour, but it also conflicts with my internal values about working with people with suicidality.” Others were not sure if the impact of this work had an impact on their client work as Sally considers, “maybe it does have an impact on the quality of my care.”

### 3.4 Perceptions of the work

Practitioners emphasized the difficult components of their jobs when they shared their experiences working with recurrent suicidal behavior. These perceptions are reflected in several sub-themes which characterize the specific nature of their work.

#### 3.4.1 The work is challenging

Working with recurrent suicidal behavior was portrayed as challenging in nature as practitioner Sally noted, “challenging is the first thing that comes to mind [with regards to this work].” Echoing this, Taylor articulated, “it’s definitely challenging.”

#### 3.4.2 The work is repetitive

Practitioners described feeling that this work was repetitive. Dee described going on ‘autopilot’, “sometimes you feel like you just become like autopilot because you are doing the same thing over and over again.”

#### 3.4.3 Feeling stuck

Conveying a sense of feeling stuck, Sally articulated, “often feeling a bit stuck; not knowing what to offer them or how to comfort them.”

#### 3.4.4 The progress can be slow

While feeling hopeful for clients’ recovery trajectory, practitioners like Abby described a sense of impatience while working toward improved outcomes:

*I would like to think they could move through the non-fatal suicidal behaviour to more helpful strategies but the clients that I work with, it’s really slow and long and maybe the impatient part of me really struggles with that.*

### 3.5 Influence of practitioner experience

The third theme uncovered the impact that practitioners’ years of experience in the field had on the impacts of this work. Practitioners noted that as their experience increased, the impacts on them reduced.

#### 3.5.1 Increasing confidence

Practitioners conveyed a sense of feeling more confident the more experience they had. For example, Kellie explained, “I feel a lot more experienced and a lot more confident when I work with people that engage in that behavior because I’ve had a lot of experience with that.” Additionally, in comparison with earlier days of practice, Mary described less self-questioning and worry as she obtained more experience. This was echoed by Frankie who noted increased confidence, particularly with assessing risk which reduced the amount of time this work flowed over into her personal life, “you probably do go home thinking about it a bit more due to lack of experience in dealing with it, not being as confident in assessing the risk compared to now.”

#### 3.5.2 Improving the management of countertransference

Mary described reduced countertransference with the more years of experience she had, “the countertransference as well is sometimes an issue, probably not so much now.”

#### 3.5.3 Coping with crisis events

Practitioners explained that their panic responses to crises reduced over time and that this was beneficial for the clients they worked with, “I’m probably a lot less reactive now than I was when I first started working in the area.”

### 3.6 Individual coping

This theme related to the various activities practitioners undertake at an individual level to sustain them in this work, including their own personal self-care practices and leaning on personal supports.

#### 3.6.1 Self-care

Engagement in self-care practices varied but included spending time with loved ones, watching television, journaling, going for walks, spending time with animals and in nature, playing music, working part-time, and pampering themselves. Practitioners spoke about seeking support from others and about not bringing work home with them, explaining that this became easier with more experience. This was noted by Mary who explained, “I used to bring that home with me a lot many years ago. Now [I] do not do that.” Others, like Molly, spoke about sustaining practices such as gaining perspective on the work:

*I also think about the ways that over the long-term, my work with these with people who are experiencing these kinds of things can be helpful to them and, you know dealing with one person who is like terrifyingly very, very unwell and very, very scary all the time. If I think about other people who [are] a bit further along in terms of their recovery trajectory, that can make that more sustaining.*

#### 3.6.2 Personal supports

Seeing a psychologist was seen as helpful as was accessing loved ones for support. This was captured by Mary as she explains, “I do talk about [clients] or kind of what’s happened to people in my life like family and friends as well, but obviously in a professional manner and it’s deidentified as well.”

### 3.7 Workplace considerations

Areas of practice perceived as important to this work were discussed by practitioners as well as the relational components of care. Practitioners further noted two important areas about their workplace: the culture and support available.

#### 3.7.1 Practice considerations

Practitioners discussed the importance of appropriate procedure including asking relevant questions about risk and ensuring they are well-prepared as Kellie explains:

*I need to be across as much information as I could possibly have about the people that we are working with that do engage in this behaviour so that we can identify the signs that they may be about to engage in that behaviour so that we can try to prevent it from happening.*

#### 3.7.2 Relational aspects

Practitioners stressed the importance of maintaining compassion for clients and ensuring the relationship is prioritized. For example, Kellie explains, “it’s just so important that we have compassion for them and it’s so important that we focus on our relationship with these clients.” However, some practitioners found it difficult to maintain empathy, as Sally notes, “that can cause me to feel guilty for being so frustrated with them.” Abby explained that maintaining the therapeutic relationship can be difficult as she focuses on managing risk instead, “I can feel myself falling into that trap of spending a lot of time assessing risk rather than really trying to work therapeutically.”

#### 3.7.3 Workplace supports

Practitioners described the types of supports available. Most practitioners acknowledged having access to the employee assistance program (EAP), however Dee felt this was inadequate. Practitioners also utilized other supports such as debriefing with seniors and/or colleagues. Practitioners discussed individual supervision although access to this varied. Supervision was almost unanimously perceived as valuable from those with access as Mary explains, “supervision is super important to me… it’s been super helpful in that space of working with recurrent non-fatal suicidal behaviour.”

Dee expressed a desire for support groups in this area, “sort of like support groups and things like that…that would be really helpful.” Additionally, group supervision was noted as important with many perceived benefits as Mary describes:

*“I think [what] should be utilised a lot more is group supervision. You get lots of different perspectives of… [what other practitioners’] perspectives are of that behaviour, how they manage it, so then you can kind of take on those little snippets of how other people manage.”*

Sally noted the value in obtaining advice from specialist services. Similarly, Dee discussed the desire for support from people who understood the work, however, it did not appear she had access to such a service:

*If there was [a] service that we can call that have had people in our positions do the same job, so that if you do call them, they understand exactly where you are coming from… Because you really need people to understand what your role entails for them to be able to assist you.*

Most practitioners expressed a need for more education and training, while some noted this used to be more readily available. Abby explained that she has not found training in this area to be adequate stating, “I have not really found a training that really explains or encapsulates or tells us how to manage non-fatal suicidal behaviour.” Molly questioned whether more training would be useful or adding to the workload.

#### 3.7.4 Workplace culture

Practitioners described workplace culture that either supports or hinders them. One concern was how the responsibility for risk is placed on individual practitioners. Frankie expressed concern about whether the new Victorian Mental Health and Wellbeing Act 2022, would exacerbate this:

*It’s terrifying a lot of people with how the Victorian Mental Health [and Wellbeing] Act [2022] is heading in the next six weeks because people are allowed to sit with higher risk, but then I feel like there’s a sense of more blame if someone was to [die by suicide] or repeatedly try and harm themselves.*

Practitioners expressed a need for an acknowledgement that this work is difficult. Some felt that this acknowledgement should come with increased remuneration while others felt a gesture from leadership would be helpful. For example, Frankie notes, “it just needs to be a bit more acknowledgement probably from management above that these presentations are really, really hard and tiring, exhausting… that acknowledgement would go a long, long way.”

More broadly, practitioners discussed qualities which they perceived as valuable from leadership. This included feeling like leadership backs them, has trust in them as well as feeling like they can tell leadership when something is impacting them so that they can take a step back. Additionally, they felt that it was important having a leadership team who both proactively checks in with staff, while also having an open-door policy.

Abby discussed the importance of sharing the load, “[there’s] only so much complexity a single person can take before it becomes overwhelming.” Molly described the importance of sharing the risk to mitigate feelings of being solely responsible for the client:

*I’ll always [bring] in other people… I find that shares the burden a little bit and it makes it feel less like I’m responsible for this person’s life or death.*

### 3.8 Systemic issues

This final theme reflected issues within systems that impacted the work. Practitioners spoke about systemic issues impacting the work making it more challenging.

#### 3.8.1 Issues impacting practitioners

Practitioners described issues with service models including having to refer actively suicidal clients out which creates ruptures in the therapeutic alliance. Additionally, practitioners described funding models that did not meet the level of demand as Abby explains, “it’s a whole system issue… we only have funding for about four full-time clinicians… to service 300 clients, it’s just not possible.”

Other practitioners discussed workforce shortages and the impact this has on their work, as Molly explains:

*The workforce is very stretched and that could mean that you feel like you do not quite get enough time, or you do not have enough mental or emotional space to work with [these] clients … and I think that’s obvious, more staff is [going to] make the work easier.*

Taylor described the impact that these shortages have on access to senior staff:

*With this kind of work, you have that high staff turnover as well. So, you have not got that that backbone of the team where you have got that that person who’s been there through it all that you can bounce ideas off.*

Practitioners spoke about the need to make this a more attractive field by offering better remuneration to practitioners. For example, Mary explains, “not so financially stressful… You have to pay for these degrees, you do lots of overtime to try and help people.” Abby explained that increasing the number of university places is important to have enough people qualified, “there’s a bottleneck, where you actually cannot get people to complete courses like for psychology.”

#### 3.8.2 Issues impacting clients

Practitioners spoke about systems not designed for this work, and a system that in some cases is harmful. For example, Abby explains, “I think in some ways, [the system] indirectly reinforces suicidal behavior, because that’s what gets a response.” Dee spoke about the options available sometimes being limited the Crisis Assessment and Treatment Team (CATT) or the police, and that these options were not always useful:

*There’s either the police or [CATT]… I do understand their frustration as well, but they’ll look at like [clients’] files and say, well, you know this person does this thing all the time, so sometimes it comes across as like they are not overly concerned because it’s a repeated action.*

Practitioners expressed a need for increased access to specialized services, psychosocial support workers and psychologists. Molly also discussed broader systems that reinforce disadvantage:

*It would be nice if my clients had secure housing and it wasn’t half of their income. Those kinds of things that would mean my work is less stressful because it feels like the people that we are looking after aren’t so lost… Potentially knowing that there’s better recourse for people who disclose trauma as well. You know, knowing that there’s things out there… to seek justice. It’s quite frustrating to say to someone, ‘Well, I’m really glad that you have felt that you were able to talk about this situation, do not go to the cops, it’ll be horrible for both of you’.*

## 4 Discussion

This is the first qualitative study exploring the experience of community mental health practitioners working with clients engaging in recurrent suicidal behavior. This study had the primary aim of understanding the impacts of working with this client population on practitioners. The secondary aim was to explore supports and resources perceived as valuable for the support and sustainability of practitioners.

### 4.1 Impacts on the wholistic self

This study mirrored previous findings about the impacts of client suicide, working with suicidal behavior and working with clients experiencing suicidal distress. Practitioners in this study described hopelessness (12), stress, anxiety and worry that it may result in suicide (20), along with increased self-questioning about their practice (21, 22). While tiredness is reported elsewhere (12), practitioners in this study reported fatigue, being drained of energy and exhaustion which may be associated with reported feelings of being activated and on edge. These may be unique to working with recurrent suicidal behavior as previous studies have not reported such impacts. Feeling activated and on edge may be related to more defensive practice which is noted elsewhere (7–9). Importantly, some practitioners perceived this work to be highly rewarding. Another noteworthy finding in this study was that one practitioner reported no psychological impacts which is consistent with previous studies whereby 9.7% of the sample in a mixed methods study on client suicide reported no impacts (23). Interestingly, practitioners in the current study spoke to the ability to see the impacts more easily in their colleagues highlighting the importance of external support for practitioners working in this space.

Discussions around work-life balance in the current study mirrored those from previous studies (20). The interruptions to work-life balance appeared to be related to needing to use rest as recovery to process the events of the week. Some practitioners described difficulties within their relationships outside of work and motivating themselves to see loved ones while others withdrew entirely. This reflected previous findings where relationships were negatively impacted because of this work (8), although their study did not provide further insights into the ways in which these relationships were impacted due to the nature of this work. The current study adds value to previous works by exploring the lived experience of practitioners to uncover specific ways in which areas of their personal lives are impacted so that targeted interventions can be established. Importantly, some practitioners explained that they left work at work, and therefore this work did not negatively impact them in their personal lives. This may be related to individual coping or potentially having access to supports to process these events during their workdays.

Practitioners noted impacts on their professional lives including irritability and anger toward colleagues for not holding the same complexity on their caseloads, as well as toward inexperienced practitioners for questioning their judgment and decision-making. Other studies also noted that some practitioners’ relationships with their colleagues were negatively impacted by this work (8), again, due to the quantitative nature of this study it is not clear in which ways relationships with colleagues have been negatively impacted and why. This study again provides insight into these impacts and the causes of this in a way that targeted interventions can be incorporated to mitigate these impacts such as processes that allow the sharing of complexity across caseloads. Consistent with previous literature, practitioners’ described changes to their work practices. These changes included: increased self-doubt (7) avoidance of clients engaging in suicidal behavior (7, 8, 23–27), seeking reassurance (8), and keeping clients at arm’s length (25). One practitioner explained that they were not sure if the impacts of this work subsequently impacted the quality of their care, while another explained that it changed the way others who were not engaging in suicidal behavior were perceived. Although while these changes were noted in practices, it was also made clear by practitioners that these did not always align with their values. Further workplace interventions should explore supports which may mitigate these changes to workplace practices to support practitioners to continue to engage in practices in line with their values and reduce the impact they have on client care.

### 4.2 Perceptions of the work

Practitioners in this study expressed feelings that this work was particularly challenging. Some of this appeared to stem from the work with clients itself, however, when interpreting this in the context of the remaining themes, much of this appeared to be related to broader systemic factors. Most practitioners in this study expressed frustration in an under-resourced and fragmented service system which did not respond adequately to these clients. Practitioners in this study described the service system as crisis-driven, and reinforcing of suicidal behavior, which is a similar finding to Victoria’s mental health royal commission (16).

### 4.3 Influence of practitioner experience

An important finding of this study was the positive impact that more years of experience in this field had on their coping. Practitioners described increased ability to manage countertransference, increased confidence and ability to cope with crises. Existing literature on client suicide uncovered conflicting results regarding the impact of experience with some studies indicating that more experience decreased the negative impacts of client suicide (28, 29), while others indicated no effect with regards level of experience (22, 24, 30, 31). This study warrants further qualitative exploration of the perceived value in increased years of experience in this field to uncover what aspects of their experience have contributed to the reduction of impacts on the wholistic self in order to incorporate these components into training and professional development for those earlier on their careers to explore whether this reduces the impacts on more junior practitioners.

### 4.4 Individual coping

Practitioners spoke about a diversity of coping skills they used to sustain them in this work. Some practitioners were able to reach out to loved ones while others withdrew entirely due to difficulties disengaging from the work. A study on working with people experiencing suicidal distress also noted difficulties disengaging from this work (20), however, given the earlier identified differences in working with recurrent suicidal behavior, further research is warranted to understand the diversity of responses in this area. These findings highlight the importance of workplace investment in the provision of supports to reduce distress among their staff so that they are able to fully participate in individual self-care practices.

### 4.5 Workplace considerations

Practitioners in the current study placed emphasis on the considerations they needed in this field of work. For example, practitioners spoke about the importance of high levels of preparation for client work and the importance of asking questions relating to risk. These findings may indicate further changes workplace behaviors to be more defensive in practice which is spoken about above and discussed elsewhere (7–9, 26, 27). Practitioners placed emphasis on the importance of maintaining empathy and compassion for this cohort of clients, however noted that this was not always easy, a similar finding to Rothes et al. (13). Importantly, practitioners discussed difficulty in maintaining a therapeutic relationship with clients which appeared related to concerted efforts to prioritize the management of risk. Further research exploring how practitioners balance the management of suicide risk while maintaining the therapeutic relationship may be worthwhile.

Practitioners in this study discussed a variety of workplace supports that helped sustain them although not all practitioners had equal access to supports and more was sought from workplaces. Several practitioners mentioned access to EAP, but no practitioner disclosed utilizing this service. Although it was evident that one practitioner in this study believed that EAP was inadequate, it was unclear from the other practitioners whether EAP was not well-appreciated and/or whether it was under-utilized. Other studies have found EAP to be under-utilized in a tertiary hospital setting, largely due to individuals being unaware of its function (32). The underutilization of EAP with practitioners was beyond the scope of the current study, but further research on exploring the value of EAP programs with this group of practitioners may be worthwhile given the considerable impacts on practitioners’ wholistic selves.

Individual supervision was perceived as highly valuable, which is reported elsewhere in the literature on client suicide and suicidal behavior (7, 11, 12). A supervisory framework has been developed for those working with suicidality (33). However, while these guidelines encourage the acknowledgement of individual reactions to the work (including emotional reactions), they do not recognize the full range of impacts which can occur (33). It is therefore recommended that supervision for those working with recurrent suicidal behavior include the normalization of the range of impacts of this work, encourage help-seeking and help source supports needed to sustain individuals in this field.

Group supervision was perceived as valuable by practitioners. One practitioner discussed the need for a support group for practitioners in this area, however, group supervision would likely suffice. Previous studies on client suicide found that support groups were unhelpful as they did not add insight which was perceived as important (24). However, as practitioners in the current study explained, group supervision offers a multitude of perspectives that may assist individual learning and may assist in developing insight into how others manage the work.

Debriefing with colleagues and/or senior staff was perceived as valuable, similar to previous findings where debriefing was perceived as helpful (8) and where health care professionals desired this following client suicide (7). The debriefing described in the current study appeared largely *ad hoc* and informal. Other studies have noted that formal debriefing can be harmful, although similarly note that access to senior staff is perceived as important after client suicide (25). Therefore, workplace supports for those working with recurrent suicidal behavior may include mechanisms for informal and ad hoc debriefing instead of formalized mandated psychological debriefing.

Consistent with other studies (13, 34), most practitioners expressed desire for more training and education. However, one practitioner considered whether this would be adding to the workload which appeared related to an inundated mental health workforce. The literature indicates that most practitioners feel ill-prepared for this work and do not feel prepared to cope with it on a personal level (8). Additionally, research on client suicide suggests that only a small number of practitioners receive training on what to expect in terms of adverse reactions and the potential impacts on themselves and their professional practice (26). Furthermore, previous research suggests that training in risk management alone is inadequate to prepare practitioners for this work and/or adverse events (9, 25). However, practitioners in this study did seek training on the management of risk with clients engaging in recurrent suicidal behavior. Given similarities between the literature on client suicide and the current study, training on what to expect in terms of adverse events and its impacts alongside of training on the management of risk in this cohort may also benefit those working with recurrent suicidal behavior. Previous research has also indicated the need for training that incorporates communicational and relational content (13). Given practitioners in this study stressed the importance of the relational aspects of care, this should also be incorporated into training programs.

Accessing specialists for secondary consultation was found to be valuable by one practitioner in this study. However, it appeared that not all practitioners had access to such a service yet felt such a service would be helpful. This study interviewed practitioners across Australia, so it is unclear what each practitioner had access to as the provision of mental health services is largely a state responsibility. However, one specialist service that can offer secondary consultation for those engaging in recurrent suicidal behavior is Spectrum, a personality disorder and complex trauma service in Victoria, Australia. However, this service is generally for those with a diagnosis of borderline personality disorder or complex trauma and is only available for general practitioners, private psychiatrists and those with clients eligible for Spectrum’s complex care service (35, 36). This limits access for smaller organizations and other private practice providers who may similarly be working with clients engaging in recurrent suicidal behavior and would benefit from secondary consultation.

Practitioners spoke about the need for non-blaming cultures where support is available from leadership. Recent literature discusses “restorative just culture,” a model which moves away from blaming cultures in the suicide prevention workforces and places the onus of providing supports to all impacted by client suicide on leadership and workplaces (37). Restorative just culture may counter negative impacts of this work, when considering the narratives of practitioners in this study on the importance of a non-blaming culture, and previous literature indicating that practitioners are more likely to be negatively impacted by client suicide if they feel blamed (21, 24). Practitioners spoke positively about workplaces cultures where they have the support of their leadership team and where risk and workloads are shared across teams. Importantly they recognized the need for leadership to acknowledge the difficult aspects of their work, as discussed elsewhere (25). The leadership and cultural factors were noted to either sustain or hinder them which is an important finding for leaders and organizations supporting this workforce.

### 4.6 Systemic issues

The final theme discussed broader system issues impacting practitioners in this field. These issues varied and practitioners noted the impact of staff shortages, workforce turnover and poor remuneration on the sustainability of this work. Furthermore, they spoke about secondary impacts including having to do overtime to make ends meet and an inability to access senior staff due to workforce turnover. While practitioners expressed the need for more staff, there was also an acknowledgement that obtaining qualified staff was impacted by bottlenecked courses like psychology where there are limited places for postgraduate courses which are a requirement for registration in Australia.

Practitioners described broader health and social services systems which reinforced disadvantage and impacted their clients. They spoke about the need for increased access to fair judicial procedures for trauma survivors and the need for secure and affordable housing. Practitioners noted a lack of access to specialist services, psychologists and psychosocial support workers and spoke about a crisis-driven system whereby in many situations they could only access the police and/or CATT which they perceived as inadequate.

Concerns about broader systems which reinforced disadvantage reflect the concerned and compassionate attitudes of the practitioners in this study. One author has asserted that the negative impacts of working in helping professions arise from feelings of injustice about clients’ situations (38). Practitioners in this study spoke about crisis-driven systems which reinforce disadvantage for clients, which likely lead to a sense of injustice among practitioners. With this in mind, self-care from diligent practitioners will likely be insufficient to counter the effects of working in unjust and overwhelmed systems. Reynolds (38) further argues that “when self-care is prescribed as the antidote, it puts the burden of working in unjust contexts onto the backs of us as individual workers” (p. 29). Previous recommendations (39) about self-care in the suicide prevention workforce may not be enough to sustain workers. The current findings indicate the importance of a systems-based lens to sustain practitioners in the workforce. This includes the promotion of self-care; the provision of workplace supports and the transformation of broader health and social systems to ensure just and equitable access to resources.

Given the diverse impacts that this work has on practitioners, it is essential that employers provide adequate support to their staff, particularly given the responsibility to eliminate and/or minimize risk as far as it is reasonably practical is the responsibility of the employer (6). Practitioners identified workplace supports such as debriefing, supervision (individual and group) along with training and education. These supports should be routinely provided by employers and targeted at the entire workforce, not simply those at perceived risk (9). Practitioners in private practice should network with other private practitioners to create individualized plans to meet their unique needs (39). The practice wisdom from practitioners in this study suggest potentially large-scale systemic work to increase supports for those engaging in recurrent suicidal behavior which will simultaneously increase the sustainability of the workforce.

## 5 Implications

The lived experience of practitioners in this study has provided valuable insights into the impacts of this work and the supports perceived as valuable to increase the sustainability of those undertaking this important work. Practitioners outlined the ways in which this work was deeply impactful on their wholistic selves, however, they also spoke of ways in which they sustain themselves, and the further supports required for sustainability in this work. Workplace supports such as individual and group supervision and debriefing, along with changes to workplace culture can be utilized by workplaces looking to enhance the wellbeing of their staff. However, further systemic action may be necessary to reduce negative impacts on practitioners such as the need for secondary consultation from specialist services along with access to psychologists, psychosocial support workers and specialist services for individual client support. Furthermore, practitioners outlined a number of other organizational responses needed to address poor remuneration, workforce shortages, staff turnover and systems which are reinforcing disadvantage. These systemic issues point to the need for widespread systemic reform to decrease the impacts on practitioners and clients alike.

## 6 Limitations

Considerations should be made when interpreting results. Practitioners in this study were all women, aged between 29–45 years old. A broader sample including other genders and an expanded age range may establish more generalizable results. Additionally, due to the snowball nature of sampling employed, sampling bias may be present as practitioners more impacted by this work may have been more likely to self-select and to identify peers with similar experiences and/or perceptions. However, it was essential that the study only engaged those with a lived experience of working with clients engaging in recurrent suicidal behavior.

## 7 Conclusion

While some practitioners indicated that they found this work rewarding, the majority of practitioners spoke to negative impacts of their work with people engaging in recurrent suicidal behavior and these impacts appeared to be cumulative. Such impacts may influence the sustainability of the workforce and impact the quality of care provided to clients. Having increased years of experience in the field appeared to reduce the negative impacts on practitioners. They spoke about helpful supports such as individual and group supervision, debriefing and secondary consultation from specialist services, although access to these supports varied. Systemic issues were perceived to impact practitioners and clients alike with difficulties accessing psychologists, psychosocial support workers and specialist services for clients. Workforce issues impacting practitioners included staff shortages and workforce turnover. As in previous studies, workforce supports were perceived to be valuable, however, practitioners in this study also stressed the need for broader systemic reform citing under-resourced and crisis-driven services that reinforced suicidal behavior in this specific cohort of clients. Additionally, they cited broader systems that reinforce disadvantage such of a lack of affordable housing and limited recourse for those who have experienced trauma. In comparison to previous studies which predominantly referred to organizational supports and individual coping, the practitioners in this study highlighted the need for broader systemic reform in both the mental health sector and broader systems that this cohort of clients has contact with. These findings suggest the need for a systems-based intervention in order to support practitioners who provide care to clients engaging in recurrent suicidal behavior.

## Data Availability

The datasets presented in this article are not readily available because this study uses a small sample of qualitative data from those with lived experience of the impacts of working with those engaging in recurrent suicidal behavior. Specifically, mental health workers’ reports of their experiences of having client/s attempt suicide and/or suicide have been disclosed in the raw data transcripts; thus the data is highly sensitive. The authors have used qualitative quotes to support the thematic reported findings to ensure the greatest privacy and anonymity of participants. Requests to access the datasets should be directed to jacinta.hawgood@griffith.edu.au.
