# Supplementary material for: Working with clients engaging in recurrent suicidal behavior: impacts on community mental health practitioners
Source: Front Public Health. 2025 Mar 13;13:1537595. doi: 10.3389/fpubh.2025.1537595 (PMC11966455; doi:10.3389/fpubh.2025.1537595)
Supplement: Supplementary file 1 [file Table_1.docx]

Supplementary Material

Supplementary Material 1

COREQ (COnsolidated criteria for REporting Qualitative research) Checklist

Developed from: Tong A, Sainsbury P, Craig J. Consolidated criteria for reporting qualitative research (COREQ): a 32-item checklist for interviews and focus groups. *International Journal for Quality in Health Care*. 2007. Volume 19, Number 6: pp. 349 – 357.

You must provide a response for all items. Enter N/A if not applicable.

| **Topic** | **Item No.** | **Guide Questions/Description** | **Reported on**  **Page No.** |
| --- | --- | --- | --- |
| **Domain 1: Research team**  **and reﬂexivity** | | | |
| *Personal characteristics* | | | |
| Interviewer/facilitator | 1 | Which author/s conducted the interview or focus group? | p.3, s. 2.2 |
| Credentials | 2 | What were the researcher’s credentials? E.g., PhD, MD | p.4, s. 2.4 |
| Occupation | 3 | What was their occupation at the time of the study? | p.4, s.2.4 |
| Gender | 4 | Was the researcher male or female? | p.4, s. 2.4 |
| Experience and training | 5 | What experience or training did the researcher have? | p.4, s.2.4 |
| *Relationship with*  *participants* | | | |
| Relationship established | 6 | Was a relationship established prior to study commencement? | p.4, s. 2.4 |
| Participant knowledge of  the interviewer | 7 | What did the participants know about the researcher? e.g., personal  goals, reasons for doing the research | p.4, s. 2.4 |
| Interviewer characteristics | 8 | What characteristics were reported about the interviewer/facilitator?  e.g., Bias, assumptions, reasons and interests in the research topic | p.4, s.2.4 |
| **Domain 2: Study design** | | | |
| *Theoretical framework* | | | |
| Methodological orientation and Theory | 9 | What methodological orientation was stated to underpin the study? e.g., grounded theory, discourse analysis, ethnography, phenomenology,  content analysis | p.3, s.2 |
| *Participant selection* | | | |
| Sampling | 10 | How were participants selected? e.g., purposive, convenience,  consecutive, snowball | p.3, s. 2.1 |
| Method of approach | 11 | How were participants approached? e.g., face-to-face, telephone, mail,  email | p.3, s. 2.1 |
| Sample size | 12 | How many participants were in the study? | p.3, s. 2.1 |
| Non-participation | 13 | How many people refused to participate or dropped out? Reasons? | p.3, s. 2.1 |
| *Setting* | | | |
| Setting of data collection | 14 | Where was the data collected? e.g., home, clinic, workplace | p.3, s. 2.2 |
| Presence of non-  participants | 15 | Was anyone else present besides the participants and researchers? | p.3, s.2.2 |
| Description of sample | 16 | What are the important characteristics of the sample? e.g., demographic  data, date | p.4, s. 3.1 |
| *Data collection* | | | |
| Interview guide | 17 | Were questions, prompts, guides provided by the authors? Was it pilot  tested? | p.3, s. 2.2 |
| Repeat interviews | 18 | Were repeat interviews carried out? If yes, how many? | NA |
| Audio/visual recording | 19 | Did the research use audio or visual recording to collect the data? | p.3, s.2.2 |
| Field notes | 20 | Were ﬁeld notes made during and/or after the interview or focus group? | NA |
| Duration | 21 | What was the duration of the interviews or focus group? | p.3, s.2.2 |
| Data saturation | 22 | Was data saturation discussed? | p.4, s. 2.4 |
| Transcripts returned | 23 | Were transcripts returned to participants for comment and/or correction? | NA |
| **Domain 3: analysis and**  **ﬁndings** | | | |
| *Data analysis* | | | |
| Number of data coders | 24 | How many data coders coded the data? | p.3, s.2.3 |
| Description of the coding  tree | 25 | Did authors provide a description of the coding tree? | p.4, s. 3.2 |
| Derivation of themes | 26 | Were themes identiﬁed in advance or derived from the data? | p.3, s.2.3 |
| Software | 27 | What software, if applicable, was used to manage the data? | p.3, s.2.4 |
| Participant checking | 28 | Did participants provide feedback on the ﬁndings? | NA |
| *Reporting* | | | |
| Quotations presented | 29 | Were participant quotations presented to illustrate the themes/ﬁndings?  Was each quotation identiﬁed? e.g., participant number | p.4, s.2.4 |
| Data and ﬁndings consistent | 30 | Was there consistency between the data presented and the ﬁndings? | p.4, s. 2.4 |
| Clarity of major themes | 31 | Were major themes clearly presented in the ﬁndings? | p.4, s. 2.4 |
| Clarity of minor themes | 32 | Is there a description of diverse cases or discussion of minor themes? | p.4, s.2.4 |
